# Correlation between preoperative CT scan of the paraspinal, psoas, and gluteus muscles and postoperative ambulatory status in patients with femoral neck fractures

**DOI:** 10.1186/s12891-024-07251-1

**Published:** 2024-02-12

**Authors:** Akihito Suto, Kengo Fujii, Takushi Nakatani, Kaishi Ogawa, Takumi Ichihara, Sayori Li, Kosuke Sato, Kousei Miura, Toru Funayama, Masashi Yamazaki

**Affiliations:** 1 Department of Orthopaedic Surgery, Showa General Hospital, 8-1-1 Hanakoganei, Kodaira, Tokyo, Japan; 2 Department of Orthopaedic Surgery, Faculty of Medicine, University of Tsukuba, 1-1-1 Tennodai, Tsukuba, Ibaraki, 3058575 Japan

**Keywords:** Sarcopenia, Femoral neck fractures, Paraspinal muscles, Gluteus muscles, Mobility limitation

## Abstract

**Background:**

This study aimed to investigate the relationship between femoral neck fractures and sarcopenia.

**Methods:**

This was a retrospective analysis of 92 patients with femoral neck fractures, from September 2017 to March 2020, who were classified into high ambulatory status (HG) and low ambulatory status (LG) groups. Ambulatory status was assessed before surgery, one week after surgery, at discharge, and during the final follow-up. To evaluate sarcopenia, muscle mass and fatty degeneration of the muscles were measured using preoperative CT. An axial slice of the superior end of the L5 vertebra was used to evaluate the paraspinal and psoas muscles, a slice of the superior end of the femoral head for the gluteus maximus muscle, and a slice of the inferior end of the sacroiliac joint for the gluteus medius muscle. The degeneration of the muscles was evaluated according to the Goutallier classification.

**Results:**

The cross-sectional area of the gluteus medius and paraspinal muscles was significantly correlated with ambulatory status before the injury, at discharge, and during the final follow-up.

**Conclusions:**

Measurement of the gluteus medius and paraspinal muscles has the potential to evaluate sarcopenia and predict ambulatory status after femoral neck fractures.

## Introduction

Femoral neck fractures, one of the most common fractures among the older adults, worsens ambulation and also affects mortality. The mortality rate after femoral neck fracture was reported to be approximately 4.0% in the short term (1–3 months) and 10–23% in one year [1–3]. Thus, the postoperative ambulatory status in the older adult population is related to mortality. Determination of the prognosis after femoral hip replacement is difficult due to individual differences, although general condition, comorbidity, age, and ambulation status before injury have been reported to affect postoperative ambulation status [1].

Sarcopenia was first described by Rosenberg in 1989 as the loss of muscle mass with age to define the progressive and generalized loss of muscle mass and strength with advancing age [4]. Currently, sarcopenia is defined as age-related loss of muscle mass, strength, and physical function. Muscle strength, muscle mass, and walking speed are all part of the diagnostic criteria [5]. The measurement of muscle cross-sectional area (CSA) and fatty degeneration has been reported to be useful in the evaluation of sarcopenia. CSA over Body Mass Index (BMI) and the Goutallier classification correlate strongly with health-related quality of life scores (HRQOLs) [5–7].

Sarcopenia has been reported to be related to mortality in older adults; however, the relationship between postoperative ambulatory status after femoral hip fracture and sarcopenia remains unknown. This study aimed to analyze the relationship between postoperative ambulatory status, muscle CSA, and fatty degeneration in patients with femoral neck fractures, which would show the importance of preventing sarcopenia.

## Materials and methods

### Subjects

From September 2017 to March 2020, 92 consecutive patients (23 males, 80.6 ± 6.7 years old; 69 females, 82.4 ± 7.2 years old) with femoral neck fractures who underwent bipolar hip arthroplasty in a single institute were included and retrospectively assessed. Out of the 92 patients, 55 were followed up for more than 30 days after discharge (Table 1). Written informed consent was obtained from all patients for publication of this study and any accompanying images.

**Table 1:** Patient demographics

		All	Follow-up period (day) ≧30 days
No. of cases		92	55
Sex (Male / Female)		23 / 69	11/44
Age (years old)		81.9 ± 7.1	83.0 ± 6.8
		Male 80.6 ± 6.7	Males 83.2 ± 5.6
		Female 82.4 ± 7.2	Females 83.0 ± 7.1
Length of hospitalization (day)		27.1 ± 12.6	27.5 ± 13.3
Follow-up period (day)		257.4 ± 270.2	411.7 ± 250.1
Ambulatory status before the injury	1	53	33
	2	16	10
	3	18	11
	4	2	0
	5	3	1
	6	0	0
Ambulatory status at 1 week after surgery	1	1	1
	2	5	3
	3	31	21
	4	21	14
	5	28	12
	6	6	4
Ambulatory status at discharge	1	4	4
	2	24	16
	3	32	21
	4	18	10
	5	14	4
	6	0	0
Ambulatory status at final follow up	1	-	33
	2	-	10
	3	-	11
	4	-	0
	5	-	1
	6	-	0
Dementia		23	13

### Radiographic measurements

**Fig. 1:**
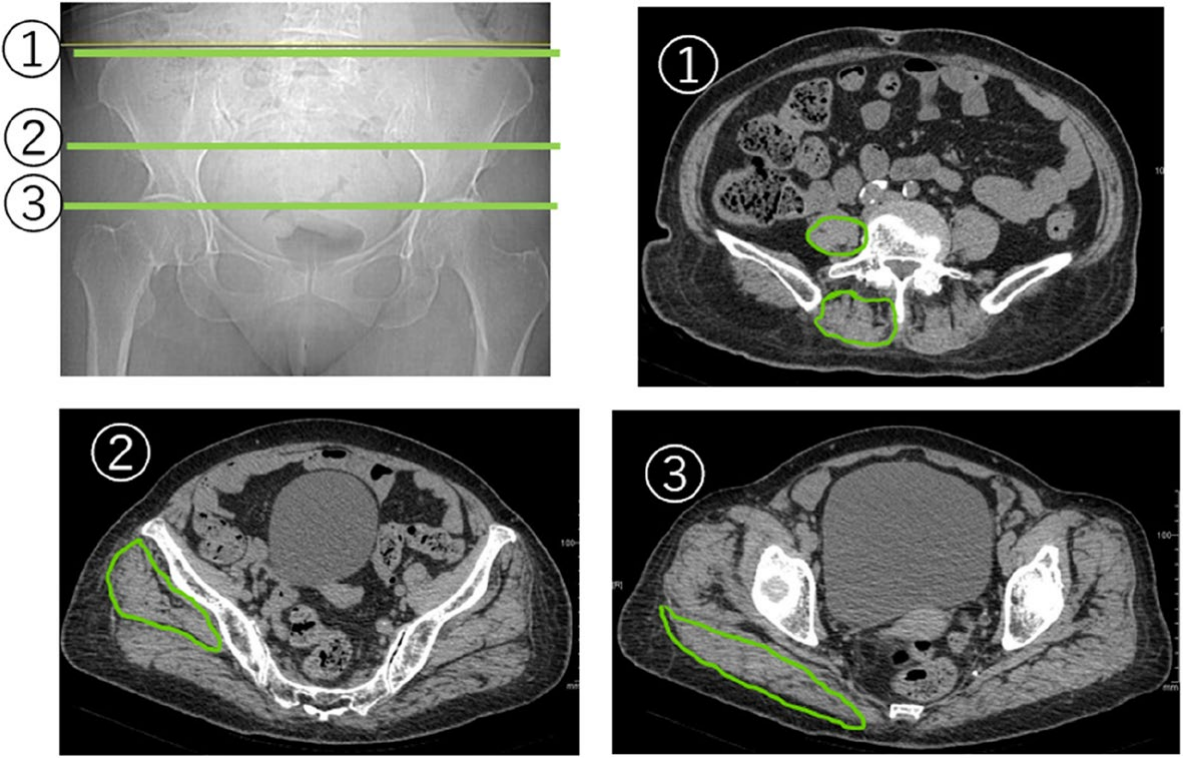
Axial CT scan slices and the measurement of muscle cross-sectional area. (1) Superior end of the L5 vertebra – paraspinous /psoas muscles; (2) inferior end of the sacroiliac joint – gluteus maximus muscle; and (3) superior end of the femoral head – gluteus medius muscle

**Fig. 2:**
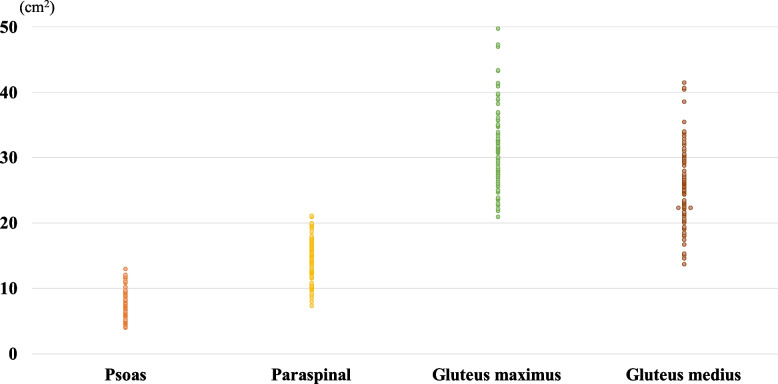
Plots of the cross sectional area (CSA) of muscles

**Fig. 3:**
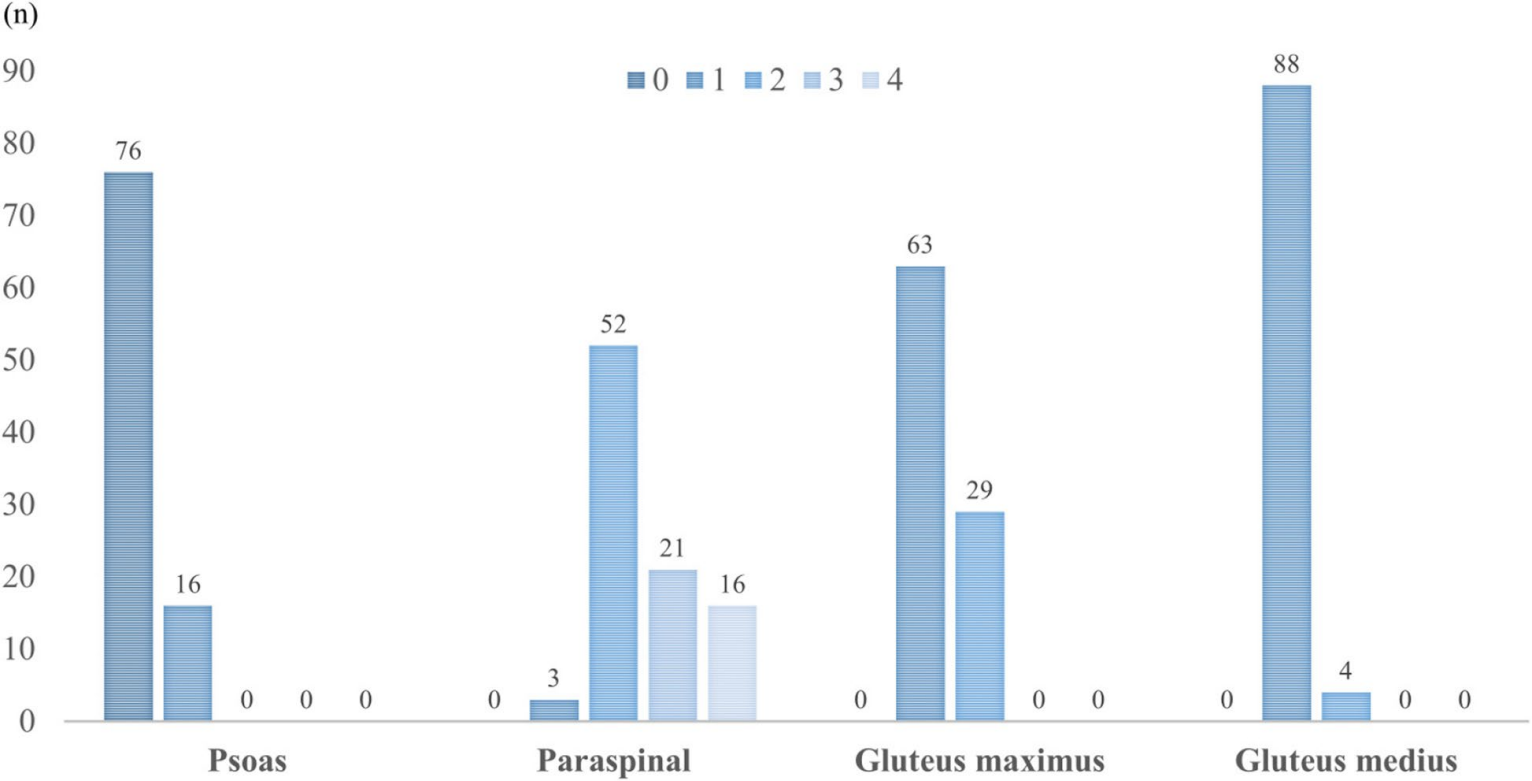
Degeneration of muscles (Goutallier classification)

Muscle CSA and fatty degeneration were assessed using a preoperative CT scan. An axial slice of the superior end of the L5 vertebra was used to evaluate the paraspinal and psoas muscles [8], a slice of the superior end of the femoral head for the gluteus maximus muscle, and a slice of the inferior end of the sacroiliac joint for the gluteus medius muscle (Table 2, Figs. 1, 2 and 3) [9]. The degeneration of the muscles was evaluated according to the Goutallier classification [10].

**Table 2:** Cross-sectional areas of muscles

CSA (cm^2^)	All (*N* = 92)	Follow-up period (day) ≧30 days (*N* = 55)
Psoas muscle	7.2	7.1
Paraspinal muscle	14.4	14.7
Gluteus maximus muscle	30.9	30.9
Gluteus medius muscle	25.5	25.6

*CSA* cross-sectional area

### Assessment of ambulatory status

Ambulatory status was assessed one week after surgery, at discharge from our hospital, and during the final follow-up. Ambulatory status was classified into the following levels: Level 1, independent walking on the surface; Level 2, walking with crutches without assistance; Level 3, use of regular front or reverse walker; Level 4, need for continuous support from one person who helps to balance and carry weight; Level 5, wheelchair; and Level 6, bedridden. The ambulatory status before the injury was self-reported by patients and their families. Abe et al. evaluated the ambulatory status after osteoporotic vertebral fractures by these 6 levels of classification, reported by Graham [11, 12]. We defined the high ambulatory status group (HG) as Levels 1 to 2 and the low ambulatory status group (LG) as Levels 3 to 6. Improvement of postoperative ambulatory status was evaluated from one week after surgery to discharge and classified into the improved group (IG) and stable group (SG).

### Statistical analysis

The paired *t*-test and Mann–Whitney U test were used for statistical analysis, and *p* values of less than 0.05 were considered to be significant. To assess the correlation of CSA at the final follow-up, regression analyses were performed. The aforementioned analyses were performed using the Bell Curve for Excel.

### Ethics approval

This retrospective study was approved by the Ethics committee in Showa General Hospital (REC-352).

### Consent for publication

Not applicable.

## Results

The distribution of CSA and degeneration of muscles are shown in Figs. 2 and 3.

**Fig. 4:**
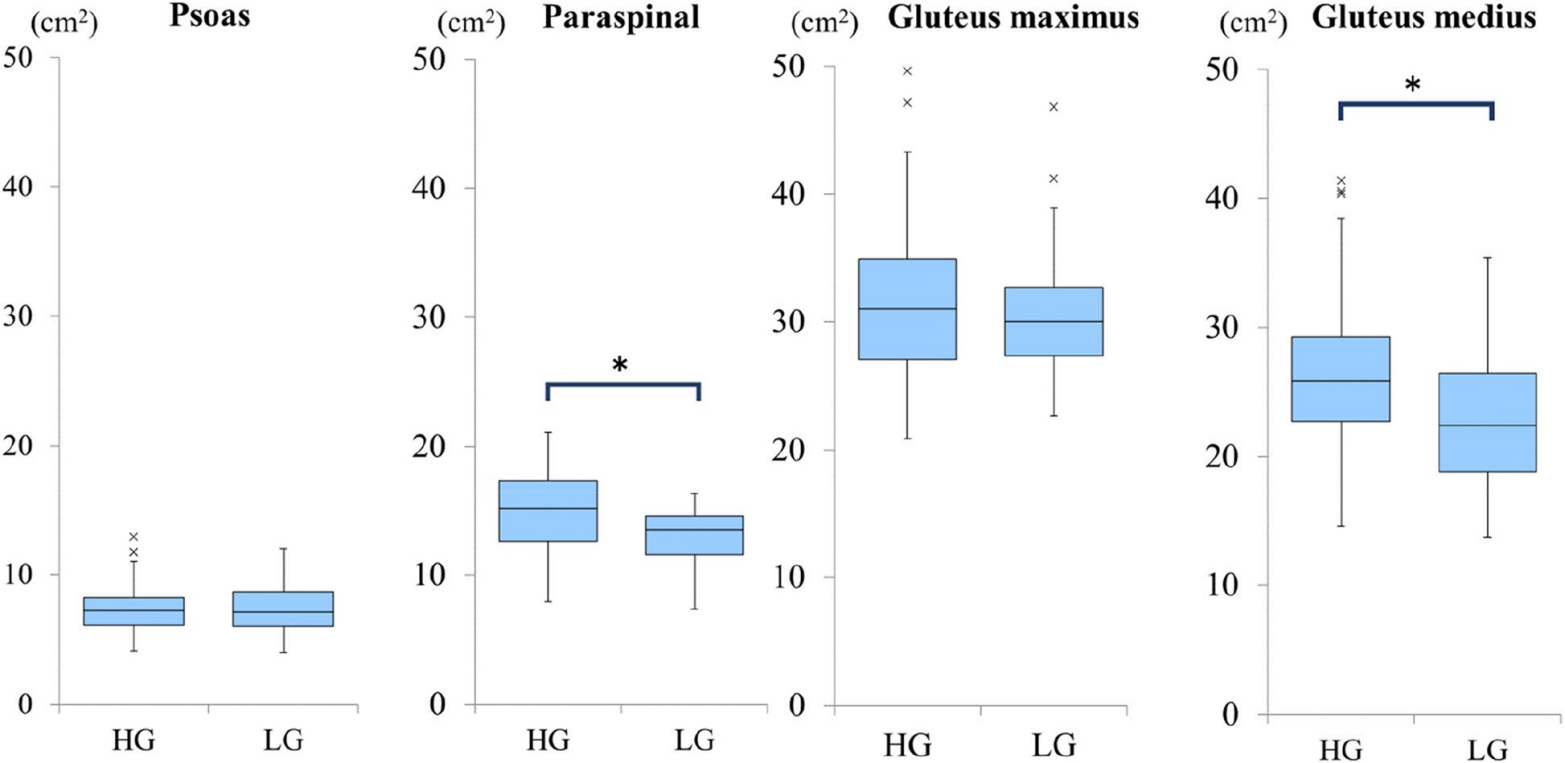
Comparison of the cross-sectional area of muscles between high ambulatory status group (HG) and low ambulatory status group (LG) before injury. HG: High ambulatory status group, LG: Low ambulatory status group, *: *p* < 0.05

Before the injury, the CSA of the paraspinal muscles (*p* = 0.01) and gluteus medius (*p* = 0.03) and fatty degeneration of the gluteus maximus (*p* < 0.001) were significantly higher in the HG. There were no significant differences in the CSA (*p* = 0.94) and fatty degeneration of the psoas muscle (*p* = 0.53) between the groups (Table 2, Table 3, Table 7, Fig. 4). At discharge, the CSA of the paraspinal (*p* = 0.02), gluteus maximus (*p* = 0.02), and gluteus medius muscles (*p* = 0.004) and fatty degeneration of the gluteus maximus muscle (*p* = 0.005) were significantly higher in the HG (Table 4, Table 8).

**Table 3:** Relationship between cross-sectional area of muscle and ambulatory status before injury

CSA (cm^2^)	High ambulatory status group (HG) (*N* = 69)	Low ambulatory status group (LG) (*N* = 23)	*P*-value
Psoas muscle	7.3 ± 1.9	7.5 ± 2.2	0.94
Paraspinal muscle	14.9 ± 3.3	13.0 ± 2.3	0.01*
Gluteus maximus muscle	31.4 ± 6.1	30.9 ± 5.5	0.68
Gluteus medius muscle	26.4 ± 5.5	23.5 ± 5.8	0.03*

*CSA* cross sectional area, *: *p* < 0.05

**Table 4:** Relationship between cross-sectional area of muscle and ambulatory status at discharge

CSA (cm^2^)	High ambulatory status group (HG) (*N* = 28)	Low ambulatory status group (LG) (*N* = 64)	*P*-value
Psoas muscle	7.7 ± 1.9	7.2 ± 2.0	0.19
Paraspinal muscle	15.6 ± 3.3	13.9 ± 3.0	0.02*
Gluteus maximus muscle	33.2 ± 6.0	30.5 ± 5.8	0.02*
Gluteus medius muscle	28.2 ± 6.1	24.6 ± 5.1	0.004**

*CSA* cross-sectional area, *: *p* < 0.05, **: *p* < 0.01

**Fig. 5:**
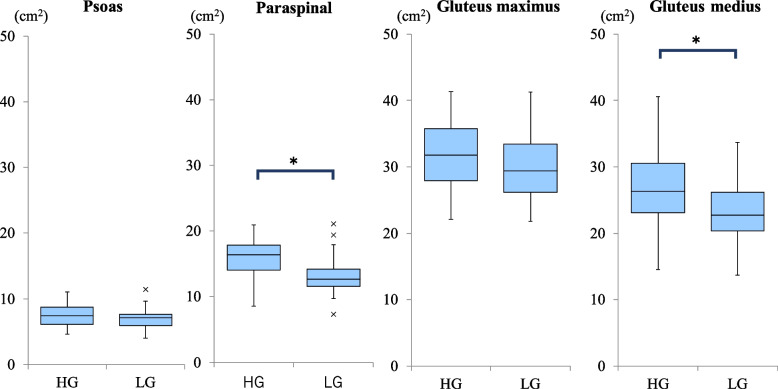
Comparison of the cross-sectional area of muscles between high ambulatory status group (HG) and low ambulatory status group (LG) at the final follow up. HG: High ambulatory status group, LG: Low ambulatory status group, *: *p* < 0.05

Among the 55 patients who were followed up for more than 30 days after discharge, at the time of the final follow-up, the CSA of the paraspinal (*p* = 0.004) and gluteus medius muscles (*p* = 0.02) and fatty degeneration of the gluteus maximus muscle (*p* = 0.02) were significantly higher in the HG (Table 6, Table 10, Fig. 5).

As for the improvement of ambulatory status from one week after surgery to discharge, the CSA of the paraspinal muscle was significantly higher in the IG (*p* < 0.001, Table 5).

**Table 5:** Relationship between muscle cross-sectional area and ambulatory status improvement from 1-week post-surgery to discharge

CSA (cm^2^)	Improved ambulatory status group (IG) (*N* = 59)	Stable ambulatory status group (SG) (*N* = 33)	*P*-value
Psoas muscle	7.2 ± 1.9	7.7 ± 1.9	0.28
Paraspinal muscle	14.9 ± 3.4	13.6 ± 2.6	< 0.001**
Gluteus maximus muscle	30.8 ± 5.5	32.2 ± 6.7	0.41
Gluteus medius muscle	25.7 ± 5.6	25.6 ± 5.8	0.89

*CSA* cross-sectional area, **: *p* < 0.01

### Case

**Fig. 6:**
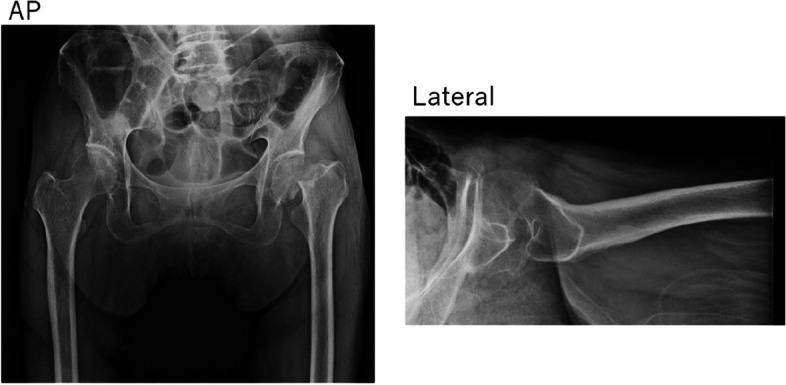
Preoperative hip plain X-rays of a representative case. (Garden classification Stage 4). Case. 81-year-old female diagnosed with a femoral neck fracture. Ambulatory status before the injury was Level 1

**Fig. 7:**
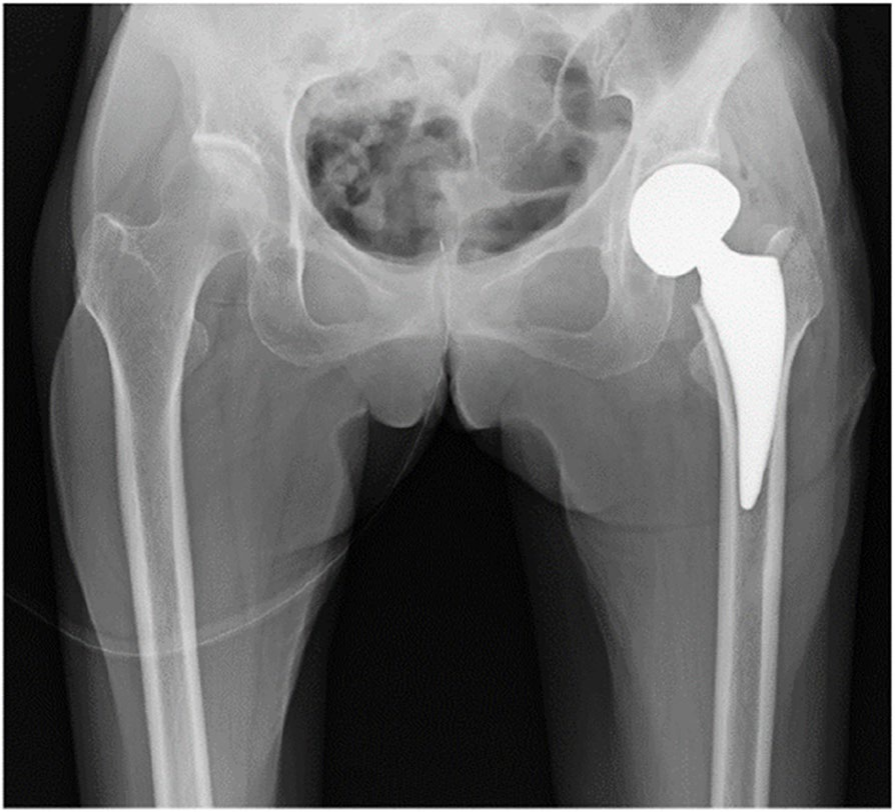
Postoperative hip plain X-rays of a representative case. Case. 81-year-old female diagnosed with a femoral neck fracture. Ambulatory status before the injury was Level 1

**Fig. 8:**
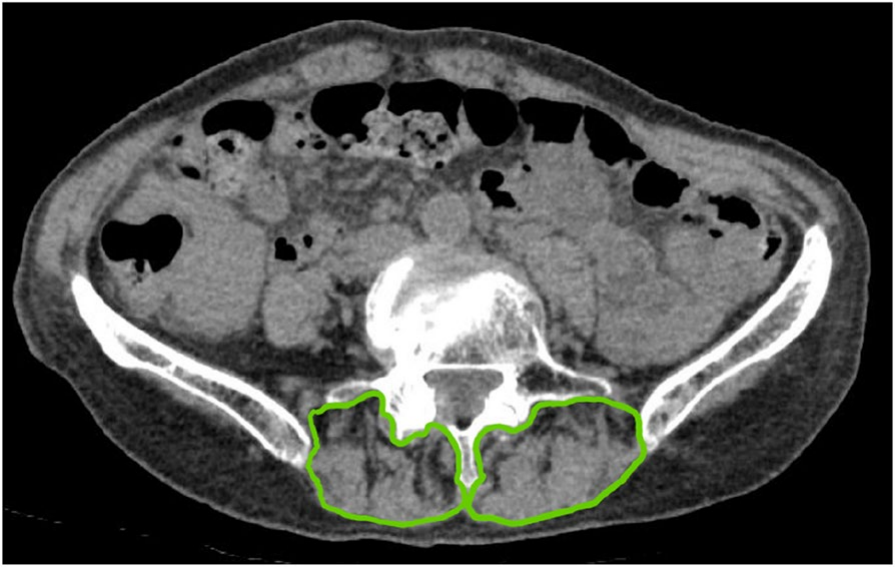
Axial CT scan slices and the measurement of muscle cross-sectional area of the paraspinous muscle. Case. 81-year-old female diagnosed with a femoral neck fracture. Ambulatory status before the injury was Level 1

**Fig. 9:**
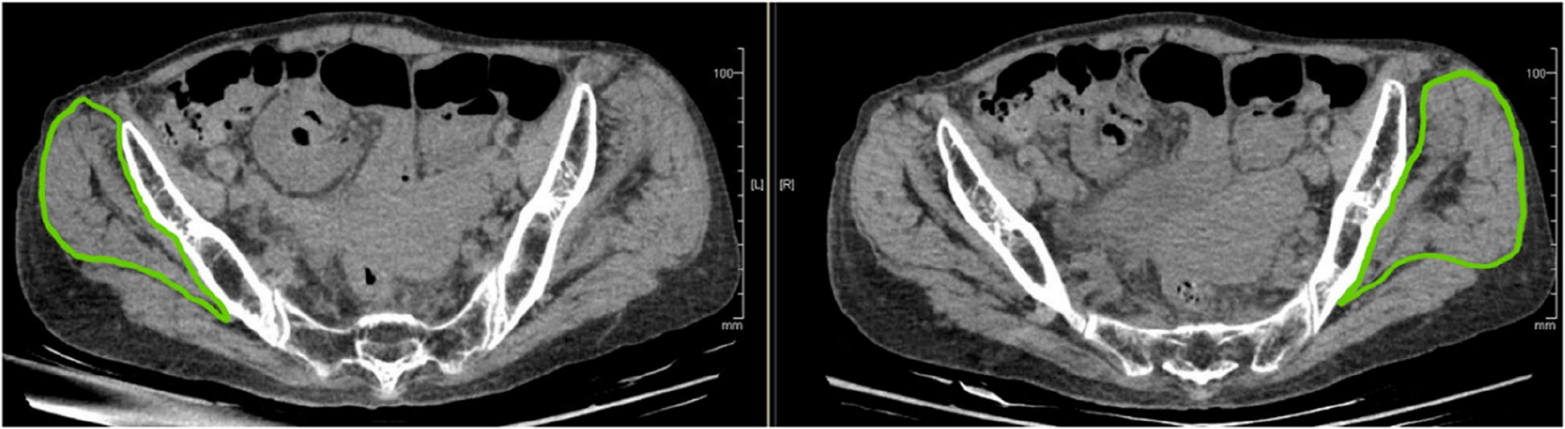
Axial CT scan slices and the measurement of muscle cross-sectional area of the gluteus medius muscle. Case. 81-year-old female diagnosed with a femoral neck fracture. Ambulatory status before the injury was Level 1

An 81-year-old female with a history of dementia and Parkinson’s disease suffered a fall. She was diagnosed with a femoral neck fracture (Garden classification Stage 4). Ambulatory status before the injury was Level 1 (Figs. 6 and 7). The CSA of the gluteus medius and paraspinal muscles assessed using pre-operative CT scans were 17.9 cm^2^ and 12.8 cm^2^, respectively, which was lower than the average CSA in this study (Table 3, 4, 5, 6, 7, 8, 9 and 10, Figs. 1, 8 and 9).

**Table 6:** Relationship between cross-sectional area of muscle and ambulatory status at final follow-up

CSA (cm^2^)	High ambulatory status group (HG) (*N* = 29)	Low ambulatory status group (LG) (*N* = 26)	*P*-value
Psoas muscle	7.2 ± 1.7	6.8 ± 1.7	0.39
Paraspinal muscle	15.9 ± 3.1	13.3 ± 3.2	0.004**
Gluteus maximus muscle	31.7 ± 5.1	30.1 ± 5.5	0.25
Gluteus medius muscle	27.4 ± 6.4	23.7 ± 5.0	0.02*

*CSA* cross-sectional area, *: *p* < 0.05, **: *p* < 0.01

**Table 7:** Relationship between degeneration of muscles and ambulatory status before injury

Degeneration of muscle	High ambulatory status group (HG) (*N* = 69)	Low ambulatory status group (LG) (*N* = 23)	*P*-value
Psoas muscle	0.16 ± 0.37	0.22 ± 0.42	0.53
Paraspinal muscle	2.5 ± 0.83	2.6 ± 0.78	0.63
Gluteus maximus muscle	1.2 ± 0.41	1.6 ± 0.50	< 0.001**
Gluteus medius muscle	1.0 ± 0.17	1.1 ± 0.29	0.24

^**^: *p* < 0.01

**Table 8:** Relationship between degeneration of muscles and ambulatory status at discharge

Degeneration of muscle	High ambulatory status group (HG) (*N* = 28)	Low ambulatory status group (LG) (*N* = 64)	*P*-value
Psoas muscle	0.18 ± 0.39	0.17 ± 0.38	0.94
Paraspinal muscle	2.5. ± 0.84	2.6 ± 0.81	0.43
Gluteus maximus muscle	1.1 ± 0.31	1.4 ± 0.50	0.005**
Gluteus medius muscle	1.0 ± 0.0	1.1 ± 0.24	0.18

^**^: *p* < 0.01

**Table 9:** Relationship between degeneration of muscles and ambulatory status improvement from 1-week post-surgery to discharge

Degeneration of muscle	Improved ambulatory status group (IG) (*N* = 59)	Stable ambulatory status group (SG) (*N* = 33)	*P*-value
Psoas muscle	0.19 ± 0.39	0.15 ± 0.36	0.67
Paraspinal muscle	2.6 ± 0.80	2.4 ± 0.82	0.08
Gluteus maximus muscle	1.3 ± 0.45	1.4 ± 0.50	0.23
Gluteus medius muscle	1.0 ± 0.18	1.1 ± 0.24	0.55

**Table 10:** Relationship between degeneration of muscles and ambulatory status at final follow-up

Degeneration of muscle	High ambulatory status group (HG) (*N* = 29)	Low ambulatory status group (LG) (*N* = 26)	*P*-value
Psoas muscle	0.21 ± 0.41	0.12 ± 0.33	0.36
Paraspinal muscle	2.7 ± 0.90	2.5 ± 0.76	0.73
Gluteus maximus muscle	1.1 ± 0.35	1.4 ± 0.50	0.02*
Gluteus medius muscle	1.0 ± 0	1.1 ± 0.27	0.13

^*^: *p* < 0.05

The ambulatory status at the final follow-up was Level 4, requiring continuous support from one person who helped to balance and carry weight.

Postoperative ambulatory status decreased by three levels from pre-injury status.

In the regression analysis, CSA of the paraspinal and age showed a significant correlation with the ambulatory status at the final follow-up (Table 11).

**Table 11:** Logistic Regression for the ambulatory status at the final follow-up

	OR	95% CI	*P*-value
Age	0.88	0.78–0.99	0.04*
CSA of Psoas muscle	0.94	0.61–1.45	0.80
CSA of Paraspinal muscle	1.30	1.02–1.66	0.03*
CSA of Gluteus maximus muscle	1.12	0.98–1.23	0.11
CSA of Gluteus medius muscle	1.03	0.90–1.17	0.69

*CSA* cross-sectional area, *: *p* < 0.05, *OR* Odds ratio, *CI* Confidence Interval

## Discussion

Muscle strength has been previously reported to correlate with muscle CSA. Sarcopenia is defined as excessive muscle mass loss with aging that impairs activities of daily living (ADL) in older adults [5]. Sarcopenia affects ADL, such as walking and standing, and may lead to the need for nursing care and increased susceptibility to falls. Sarcopenia has also been reported to affect the severity and mortality of other diseases and is currently being focused on by a variety of medical specialties [13–15].

In general, femoral neck fractures reduce the ambulatory status. Various factors, such as end-stage renal disease, cirrhosis, cerebrovascular disease, and pre-fracture ambulatory status, have been reported as predictors of postoperative ambulatory status.

Regarding the relationship between femoral neck fractures and sarcopenia, the American Society of Anesthesiologists Physical Status classification system was reported as an independent prognostic factor [1, 16].

However, few studies have previously reported on the relationship between sarcopenia and the postoperative ambulatory status of femoral neck fractures. Jung et al. reported that muscle mass in the hip flexors decreased after surgery for hip fractures [17]. Recently, the serum creatinine to cystatin C ratio was reported to reflect preoperative and early postoperative walking ability in older patients with hip fracture [18].

Muscle CSA and fatty degeneration were assessed using preoperative CT slices. A CT scan is routinely performed for femoral neck fractures as a preoperative evaluation allowing us to assess the muscle without additional examination.

In summarize of our results, first, the CSA of the paraspinal and gluteus medius muscles correlated with pre-injury ambulatory status (Table 3, Fig. 4). According to these results, the CSA of the paraspinal and gluteus medius muscles can be used to identify sarcopenia.

Second, the CSA of the paraspinal, gluteus maximus, and gluteus medius muscles correlated with ambulatory status at discharge (Table 4), and the CSA of the paraspinal and gluteus medius muscles correlated with final ambulatory status (Table 6, Fig. 5). In the regression analysis, age and CSA of the paraspinal muscles showed a significant correlation with final ambulatory status (Table 11). These results seem reasonable and consistent with actual clinical practice. These suggest that the assessment of muscle CSA using preoperative CT could be useful in predicting postoperative ambulatory status.

Third, the improvement in ambulatory status from one week after surgery to discharge was significantly correlated with the CSA of the paraspinal muscle (Table 5). Onuma et al. reported the activities of the gluteus medius muscle on the stepping side and paraspinal muscle on the stance side prior to the onset of movement in older adults. However, no activity was observed in the paraspinal muscles of young adults at the onset of movement [19]. The action of the paraspinal muscles for trunk stabilization during walking in older adults might support and explain our results.

Kuno et al. reported that even in older adults, muscle strength training increases muscle mass. However, they also mentioned that aerobic exercise alone, such as walking, does not increase muscle mass [20]. Thus, postoperative ambulatory status could potentially be improved by increasing the muscle CSA, muscle mass, and muscle strength through effective rehabilitation.

In future studies, muscle strength measurements should be performed before and after surgery. Moving forward, a more aggressive rehabilitation regimen should be considered as a therapeutic intervention to increase muscle strength. Comparing the muscle strength and CSA between aggressive and normal rehabilitation will be the next step in solving our hypothesis.

Adequate therapeutic intervention after adjusting the workout intensity for individuals could potentially improve the postoperative ambulatory status after injuries such as femoral neck fractures. We believe that the results and insights from this study will help manage patients.

This study had a few limitations. First, we used the muscle CSA to evaluate muscle volume without correcting for the patient’s habitus. Previous studies have included corrected values by dividing CSAs by BMI. We did not examine height and body weight in this study. Second, the patient’s position on the CT scan and pelvic alignment might have impacted the variance of the CSA in axial slices. Other methods to directly measure muscle volume should be considered to improve the accuracy of evaluation. Third, there is variation of plots of the cross sectional area among patients in each muscle (Fig. 2). Regarding degeneration of muscles, in contrast that the psoas muscle and the gluteus medius muscle had less degeneration of muscles, there is variation of the paraspinal muscle and the gluteus maximus muscle among patients (Fig. 3). The Goutallier classification is a qualitative grading system for evaluating fatty infiltration, degeneration, and atrophy of muscles. We would like to select the appropriate muscle to evaluate, and quantitative evaluation of the muscles should be considered to obtain a more accurate analysis.

## Conclusions

The measurement or evaluation of CSA using preoperative CT may be useful in predicting improvement and postoperative ambulatory status after bipolar hip arthroplasty for femoral hip fractures in older adults.

## Data Availability

The datasets used and/or analysed during the current study are available from the corresponding author on reasonable request.
